# Preoperative and Perioperative Predictors of Length of Hospital Stay after Primary Total Hip Arthroplasty—Our Experience on 743 Cases

**DOI:** 10.3390/jcm10215053

**Published:** 2021-10-28

**Authors:** Rocco Papalia, Biagio Zampogna, Guglielmo Torre, Giuseppe Francesco Papalia, Ferruccio Vorini, Marco Bravi, Erika Albo, Antonio De Vincentis, Vincenzo Denaro

**Affiliations:** 1 Department of Orthopedics and Trauma Surgery, Campus Bio-Medico University of Rome, 00128 Rome, Italy; r.papalia@unicampus.it (R.P.); b.zampogna@unicampus.it (B.Z.); g.papalia@unicampus.it (G.F.P.); f.vorini@unicampus.it (F.V.); e.albo@unicampus.it (E.A.); denaro@unicampus.it (V.D.); 2 Multi-Specialist Clinical Institute for Orthopaedic Trauma Care (COT), 98124 Messina, Italy; 3 Department of Physical Therapy and Rehabilitation, Campus Bio-Medico University of Rome, 00128 Rome, Italy; m.bravi@unicampus.it; 4 Department of Internal Medicine and Geriatrics, Campus Bio-Medico University of Rome, 00128 Rome, Italy; a.devincentis@unicampus.it

**Keywords:** total hip arthroplasty, comorbidity, hospitalization, renal function, glomerular filtration rate

## Abstract

The aim of this retrospective investigation is to evaluate the correlation between several preoperative and perioperative factors and the length of hospital stay in patients that underwent elective total hip arthroplasty with overnight admission. Medical records of patients that underwent THA from the beginning of 2016 to the end of 2018 were retrospectively screened. Demographics, comorbidities, whole blood count, intraoperative details, and length of postoperative stay were retrieved. The association between clinical, laboratory and surgical factors and the length of hospital stay was explored by means of negative binomial and logistic regression models. The median length of postoperative hospital stay was four days (Inter Quartile Range, IQR 3, 5). After univariate regression a stepwise multivariate regression showed that operative time (*p* = 0.001), the preoperative serum creatinine (*p* < 0.001), the intraoperative blood loss (*p* = 0.04) and the use of an anterolateral approach (*p* < 0.001) were found to correlate significantly with the increase of the hospitalization length, while no significant correlation was found for all the other features. Multivariable model fitted through logistic regression (LOS below or over the median value of four days) had an Area Under the Curve (AUC) of 0.748. Our analysis suggests a significant role played by different preoperative and perioperative variables in influencing the length of hospital stay.

## 1. Introduction

Total hip arthroplasty (THA) represents the gold standard for the elective treatment of symptomatic degenerative and rheumatologic diseases of the hip at later stages [1]. It is considered as one of the most successful procedures in orthopedic surgery, as good functional and clinical outcomes have been reported in literature for almost all patient populations [2]. It is considered a major procedure regarding its invasiveness and surgical complexity, although a high number of procedures per year is reported worldwide. According to national registries, over 100,000 hip arthroplasties are carried out each year in Italy [3], with an estimated increase in the request for THA of 174% by 2030, doubling the number of revision arthroplasties by 2026 [4,5]. Given the high volume of THA and the need to manage the patient at the best medical standards, there is a need to accurately understand preoperative and perioperative clinical factors, to improve recovery and limit complications. Although in the USA and UK many surgeons perform THA in a day-surgery setting, in several European countries, the hospital admission is still necessary to optimize general patient status, especially in the elderly, before discharge [6]. After the surgery, the patient stays in the orthopedic department for a total of 2 to 4 days, to have administered pain control therapy, start rehabilitation program and control general status. If a postoperative complication occurs, recovery length may increase up to 10 days [7]. As soon as the patient is stable in regard to general status, and pain is controlled, he or she can be discharged to a rehabilitation ward or facility [8,9]. Alternatively, if functional status is definitely good, the rehabilitation protocol can be continued with supervised exercises either at patient’s home or as an outpatient [10]. In the perioperative period, there is a plethora of factors that must be considered to provide the patient with the best prognosis. These factors include comorbidities, the age of the subject, emotional status and psychological factors, thromboembolic events, postoperative fever, anemia, anesthesiologic factors [11,12,13,14,15]. However, the major factors that influence surgical success can be divided into individual factors (patient-based, including age, comorbidities, renal function, general parameters, psychological factors) and surgical factors (blood loss, use of cement, surgical approach, and duration of surgery). In recent studies [6,13,15,16,17,18] it has been reported that there are some independent factors that influence the length of hospital stay, including chronic heart failure, sepsis, bleeding disorders, and preoperative laboratory values of sodium, white blood cells and albumin. In the present literature, a focus has been put on the role of chronic kidney disease (CKD), as a major factor influencing the postoperative outcomes of THA, with several trials investigating the results of patients affected by CKD under dialysis or therapy [19,20]. However, according to our knowledge, no study investigated preoperative and perioperative factors in multivariate models, to assess the role of unique factors in predicting an increased length of hospital stay. Especially for blood loss, use of cement, day of surgery and surgical time, low evidence is available in cohorts of primary elective THA patients [17,18].

The main aim of the present study is to investigate the potential correlations of preoperative and perioperative factors with the length of hospital stay in the orthopedic ward, for subjects that underwent elective primary THA. A secondary aim is to develop and evaluate a multivariate model to predict the risk of increased LOS. The null hypothesis is that none of the evaluated factors shows a correlation with the length of hospitalization.

## 2. Materials and Methods

### 2.1. Study Design

A retrospective evaluation of electronic medical records at Campus Bio-Medico University Hospital, in Rome, Italy, was carried out. The STROBE statement was used to check for a correct reporting. Medical records of patients that underwent THA since the 1 January 2016 to the 31 December 2018 were screened. The study was approved by the Ethical Committee of the Institution (Protocol number: 32/19 OSS ComEt CBM). Informed consent for the treatment of personal health information for research purposes was signed by all patients at the first registration at our institution. The study was conducted in respect of the principles of the declaration of Helsinki for research on human subjects.

### 2.2. Population

**Figure 1:**
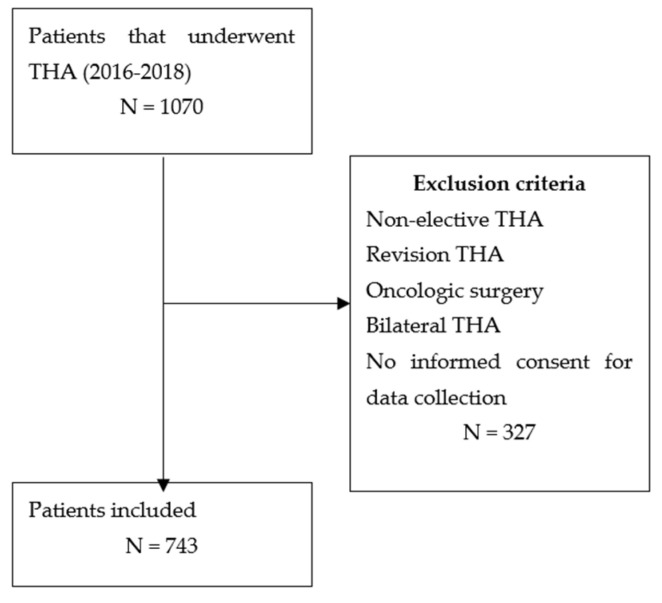
Medical records selection flow-diagram.

Inclusion criteria were adult subjects that underwent elective primary THA surgery; diagnosis of primary or secondary osteoarthritis; medical record available within electronic database of the institution; complete records including all the clinical data of the patient. Exclusion criteria were the following: revision surgery, hip fracture, oncologic patients, one-stage bilateral procedures, no informed consent given for data collection. All surgeries were carried out at our institution by two experienced surgeons, with a minimum 20 years of experience in adult joint replacement. Selection process is shown in Figure 1. All the patients underwent the same perioperative thromboembolic prophylaxis with bilateral elastic stockings, low molecular weight heparin, dosing according to weight. Furthermore, the standard antibiotic prophylaxis with II generation cephalosporin was administered (2g preoperative, 1g every 5 h for three times, postoperatively). For those with known hypersensibility reaction to penicillin and cephalosporins, Clindamicin (500 mg before the surgery and 500 mg 12 h after surgery). All patients were discharged to a rehabilitation facility, as soon as they were able to walk with two crutches (allowed from the second postoperative day), and postoperative pain was controlled. Furthermore, the hemoglobin (Hb) level was checked daily, to ensure the patient was discharged with Hb within the normal range.

### 2.3. Data Extraction

Of the data available in the medical records of the included patients, after accurate review of the literature, consensus was reached within a multidisciplinary team of investigators (G.T., R.P., B.Z., A.D.V., and M.B.) upon which data should be included in the analysis. Demographic data included sex and age, and usual residence of the patient. Among comorbidities, patient’s records were screened for presence of diabetes mellitus, arterial hypertension, dyslipidemia, heart failure, coronary disease, Chronic Obstructive Pulmonary Disease (COPD), Chronic Kidney Failure (CKF). The American Society of Anesthesiologists (ASA) score was also extracted. Furthermore, information about smoking habits were retrieved (smokers or smoke in the past). Preoperative whole blood count and inflammatory markers (Erythrocytes Sedimentation Rate, ESR, and C-Reactive Protein, CRP) data were also collected. Surgical details included: surgical approach, operative time, use of cement for cup and/or stem, and intraoperative blood loss. Data on the length of postoperative orthopedic ward stay were retrieved.

### 2.4. Data Processing

The LOS was processed as a discrete variable (days of stay after the surgery), obtained by the difference in nights between the date of discharge and the day of surgery. LOS was also transformed into a dichotomous variable, where the median value of the discrete series was used as a cut-off to identify a short (minor or equal to the median) or long (major than the median) stay. Data on usual residence of the patient have been collected to assess whether the patients lived inside or outside the city. Living outside the city (within the region) and living outside the region were processed as two binary (dichotomous) variables. Day of the week of the day of surgery was also found, through the processing of the date of surgery, the series was then transformed from a discrete to a dichotomous one, where “Friday” and “Other weekdays” were the binary values. Estimated Glomerular Filtration Rate (eGFR) was calculated through the Chronic Kidney Disease Epidemiology Collaboration (CKD-EPI) method [20,21]. The Charlson Comorbidity Index (CCI) [22] was computed to represent the global burden of comorbidities for each patient. This index was processed as a discrete five-levels variable and as a dichotomous variable with a cutoff value of 1, consistently with previous literature [23].

### 2.5. Statistical Analysis

Database consistency and completeness was assessed before proceeding with the planned analysis. Data series had a maximum incompleteness of 8%. Missing data in continuous series were addressed through the imputation of the mean, in discrete or binomial distribution, the median was used for imputation. Continuous variables were shown as mean with standard deviation (SD), while discrete variables through the median and interquartile range (IQR). Categorical Binomial variables were presented as absolute numbers and percentages. Length of hospital stay after surgery was defined as dependent variable, distribution and variance were checked, to choose the best regression model. The association between clinical, laboratory and surgical factors and the length of hospital stay was explored by means of multiple univariate negative binomial regression models and expressed as Incident Risk Ratio (IRR) with 95% confidence intervals (95%CI). A multivariable negative binomial model was also fitted including as independent variables all factors found associated with *p* < 0.10 at univariate analysis. Stepwise selection with backward deletion was carried out, to define a model with all-significant independent predictors. The best-fit negative binomial multivariate model was subsequently fitted in logistic regression, using the dichotomic transformation of LOS as dependent variable. The output was reported as Odds Ratio with 95%CI. A Receiving Operator Characteristics (ROC) curve was plotted and Area Under the Curve (AUC) was calculated. Youden’s index was then calculated to find the cut-off values for continuous predictors. Tabular analysis was carried out to found discriminants in discrete predictors. The significance threshold was conventionally set with *p* < 0.05. All analyses were conducted with STATA software for statistical computing for Mac (version 12, StataCorp, Texas, USA).

## 3. Results

### 3.1. Demographic and Anthropometric Data

A total of 743 patients satisfied inclusion and exclusion criteria and their data were collected and analyzed. The mean age was 68.1 years (SD: 10.4) and 333 subjects (44.8%) were male. Patients living outside the city, but within the administrative region were 378 (51.3%), while those living outside the region were 107 (14.5%) (Table 1).

### 3.2. Comorbidities

The most commonly observed comorbidity was arterial hypertension (47%), followed by dyslipidemia (20.4%), chronic kidney disease (12.1%) and diabetes mellitus (9%). Details on comorbidities prevalence were reported in Table 1. The CCI had a median value of 3 (IQR 3, 4), while ASA score had a median value of 2 (IQR 2,2). Details on CCI and ASA in the whole cohort are summarized in Table 2.

### 3.3. Surgery-Related Factors

The decubitus and surgical approach varied within the cohort, with the lateral decubitus, direct lateral approach being the most common (42.2%), followed by the supine decubitus, anterolateral approach (35.7%). Details are summarized in Table 3. Duration of surgery (operation time) averaged 78.3 min (SD: 13.4). Intraoperative blood loss averaged 286.2 mL (SD: 156.8). Details are reported in Table 1.

### 3.4. Multiple Univariate Regression Analysis

The discrete LOS series approximated Poisson distribution, with large overdispersion. Thus, the negative binomial model was used to fit regression. By fitting multiple univariate models for each independent variable, those significantly associated to the risk of increased LOS were: the presence of Chronic Kidney Failure (CKF) (IRR = 1.18, 95%CI = 1, 1.4, *p* = 0.041), an increased value of preoperative white blood cell (IRR = 1, 95%CI = 1,1, *p* = 0.038), increased preoperative creatinine value (IRR = 1.29, 95%CI = 1.01, 1.67, *p* = 0.041), increased operative time (IRR = 1.003, 95%CI = 1, 1.004, *p* < 0.001), increased intraoperative blood loss (IRR = 1.001, 95%CI = 1, 1.001, *p* < 0.001) and the use of a supine decubitus, anterolateral approach (IRR = 1.25, 95%CI = 1.16, 1.36, *p* < 0.001). Conversely, the variables significantly associated to a reduced LOS were limited to the inguinal approach (IRR = 0.86, 95%CI = 0.77, 0.98, *p* = 0.02) and lateral decubitus, direct lateral approach (IRR = 0.83, 95%CI = 0.77, 0.9, *p* < 0.001). Full coefficients summary is reported in Table 4.

### 3.5. Multivariate Regression Analysis

**Figure 2:**
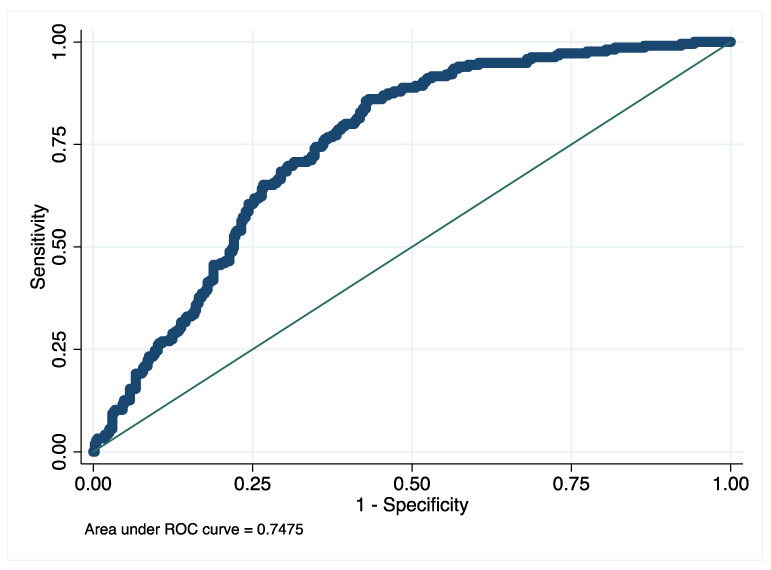
ROC curve of multivariate logistic model.

To investigate the goodness of predictors individuated through the univariate analysis, a stepwise forward-back process was used to fit the best multivariate negative binomial model. As a result of the process, a model was fitted using the preoperative creatinine value, the operation time, the intraoperative blood loss and the use of a supine decubitus, anterolateral approach (as shown in Table 4). The model had a Likelihood Ratio (Chi^2^) of 70.2. All independent variables in the model had a significant association with an increased LOS (*p* < 0.05 for all, see Table 4 for details). The same model was fitted using logistic regression with the dichotomous LOS (short stay = below or equal to 4 days, long stay = over 4 days of stay) as dependent variable. The model showed a Likelihood Ratio (Chi^2^) of 97.5. ROC curve was plotted for the logistic model, showing an AUC of 0.748 (Figure 2). Youden’s index (Yi) was computed, to find the cut-off values of continuous predictors. The Yi was 0.416 and the cut-off values were 0.9 mg/dL for creatinine, 500 mL for intraoperative blood loss, 89.2 min for the operative time (Table 5). 

## 4. Discussion

Several preoperative and postoperative factors that influence the length of hospital stay after elective primary THA were investigated in the present study. Results of this large-sample retrospective analysis showed that the factors most significantly correlated to the length of hospital stay were the duration of surgery, the intraoperative blood loss, the preoperative creatinine, and the use of an anterolateral surgical approach. All of these factors had a relevant odds ratio in predicting a LOS over the median of 4 days and the regression model generated a ROC with an AUC of 0.748.

Primary total hip replacement represents a popular and successful surgery, since good outcomes are reported in several studies worldwide [2,22]. Beside surgical techniques development, a concrete focus has been put on perioperative clinical features and general management of the patients and their cost to the health system. In a perspective of attention to medical expense, with special care for fast-track procedures and prompt recovery after major surgery, a comprehensive knowledge about factors that influence the time the patient spend inside the hospital is mandatory. In a recent review it has been reported that THA in Caucasian patients aged 85 years or more, had an average lifetime cost of $9100 higher than those for patients treated conservatively [24,25]. Another study [18] compared hospital costs for patients discharged with an Enhanced Recovery After Surgery (ERAS) program, reporting that this was $2900 higher for patients with a LOS of 2 days, compared to those with 1-day admission [18]. Therefore, an occulated containment of the costs should be considered, always preserving the safety and wellbeing of the patient.

It has been demonstrated that the presence of comorbidities and of postoperative complications are independently associate to the increase of total medical cost for the single patient [26]. More specifically, for THA a review of the literature showed that there is evidence associating the total comorbidity burden to an increased LOS [27]. In several studies [17,18,22,23,28,29] a higher CCI were found to increase the LOS, with measures of risk ranging from a Relative Risk of 1.1 [28], to an OR of 6.22 [17]. These results are attributed to a lower general functional status of patients with systemic comorbidities [27], which requires a longer postoperative period to achieve the full walking ability with crutches, without therapist aid or supervision. Furthermore, the incidence of early postoperative complications is higher in patients with compromised general health. Consistently with these studies, our results showed a significant association of a CCI > 1 with an increased LOS (OR = 1.19) at univariate analysis; however, this significance was lost in stepwise multivariate model, thus showing that the CCI is not independently associated to a longer LOS. This result is in contrast with previous literature, as only logistic univariate analysis was carried out in similar studies, predicting a binomial target of shorter or longer hospitalization. Furthermore, individual comorbidities were not associated to an increased LOS, except for CKF. These results are in concordance with a recent study by Ding et al. [30]. In a previous study by Sibia et al. [18], results showed that coronary disease was associated positively with an increased LOS. However, in this study, the cut-off value for increase in LOS was 2 days, as an ERAS program was followed for discharge of patients. In our study, the increase in preoperative creatinine value was significantly associated to increased LOS, consistently within the regression models, with a cut-off value of 0.9 mg/dL in the prediction of a LOS longer than 4 days. This could be clinically explained that pain control is more complex for patients with kidney disease and increased preoperative creatinine, as the use of NSAIDs in these patients should be limited. Some recent investigations are consistent with our findings in assessing the role of renal function in influencing the length of hospital stay for joint arthroplasty surgery [19,20,31]. Moreover, the eGFR has been advocated as a useful tool in stratifying the risk of complication in total hip and knee arthroplasty [21]. The studies evaluating the role of CKD and eGFR decrease in patients undergoing THA showed significant results that suggest a major role of this comorbidity in the development of postoperative complications [32,33], infections [33], and in the increase of postoperative length of hospital stay [20].

Operative time, intraoperative blood loss and surgical positioning and approach are the intraoperative factors found to be statistically associated to a prolonged LOS. However, although statistically significant, the association of operation time and intraoperative blood loss was definitely slight, with a respective IRR of 1.002 and 1.003 (OR of 1.01 and 1.001, respectively). Given the observed result, these factors could be considered not clinically relevant in defining a real-world risk factor for an increased LOS. However, an increased intraoperative blood loss may lead to an increased likelihood of blood transfusion, thus the need for a prolonged stay. Unfortunately, data on blood transfusion were not available in our database. These results may put the basis for further investigation concerning intraoperative factors influencing THA outcomes and postoperative LOS. Consistently with this observation, in a paper by Abbas et al. [17], no association was found between the operative time and LOS in patients undergoing THA (*p* = 0.154) [17].

A recent systematic review [34] reported that studies investigating the LOS for patients treated with different surgical approaches showed a difference between groups lower than 1 day of stay. The study concludes that there is no evidence that direct anterior approach is superior to posterolateral or lateral approaches to THA [34]. According to our results, the use of anterolateral approach was consistently and significantly associated with increased LOS (OR = 3.9), compared to the other approaches. Furthermore, the posterolateral and inguinal approaches showed a protective effects in terms of prolonged LOS (IRR of 0.86 and 0.83 respectively), though limited to univariate analysis. A previous investigation [18] showed an increased LOS for patients approached through posterolateral technique, with an OR of 3.5 [18]. However, also in this case, only univariate regression was carried out and the authors acknowledge that debate is still active on this issue.

Multivariate models have been rarely investigated in this field. A study by Roger et al. showed that no comorbidity reached a relevant hazard ratio to postpone the discharge after the 5th postoperative day [6], though only logistic model was fitted and a high cut-off value (5 days of LOS) was considered for binary transformation of hospitalization length. Furthermore, no measures of goodness-of-fit was reported [6]. Another recent investigation [30] showed that only female gender, diagnosis of primary hip osteoarthritis, and living alone condition were independently associated to a LOS longer than 3 days, in an ERAS program cohort [30]. Female gender has been reported in other studies [17,18] to be significantly associated to increased LOS and this was attributed to a decreased functional status of women compared to men in the preoperative period [18]. However, the studies had a smaller cohort than our one, thus statistical power may underestimate the results. According to our knowledge, no previous study reported the AUC of a multivariable model as a measure of goodness. While most of recent investigations [35,36,37] focused on machine learning algorithms for the definition of predictors for increased LOS, we believe that variable selection and model development should begin from accurate classical statistical modeling, paying attention to dependent distribution in real-world datasets. In fact, in recent literature, only the paper by Tabatabai et al. [38] used a negative binomial regression for duration of hospitalization, acknowledging that LOS in days has a discrete distribution with overdispersion, thus different from classic Poisson distribution.

The major strength of the present study is the large cohort we reviewed, to achieve clinical relevance and external validity. The results of our investigation are partially in line with those reported in literature, although a few studies reported data of such a wide cohort and no study fitted more than two appropriate models to individuate independent predictors for increased LOS. Furthermore, we investigated all the preoperative and perioperative factors potentially relevant for the duration of stay, according to existing literature, thus preventing selective reporting. However, the study is not free from limitations. Firstly, we limited our analysis considering the orthopaedic ward stay and did not have data on the postoperative rehabilitation time at the rehabilitation facility. Furthermore, no data on functional status of patients before and after surgery and no data on complication were available. Moreover, the retrospective design and the absence of a single surgeon performing the surgeries may be relevant bias for the clinical inference of these results.

Future research in this field should be focused on the role of non-steroidal anti-inflammatory drugs (NSAIDs) in influencing the renal homeostasis, with possible drawbacks on progressive kidney failure for those patients that have NSAIDS self-administered in preoperative period, due to the severe ongoing arthritis. Furthermore, there is a lack of data and evidence concerning the role of social and family support for these patients [39].

## 5. Conclusions

In conclusion, among the several factors that influence the length of hospital stay after THA, our analysis demonstrated a significant role played by the preoperative creatinine (as an index of renal function) and of anterolateral approach. Particular attention should be directed to patients with already established CKD at hospital admission, especially concerning increased creatinine and NSAIDs administration in these subjects. The role of surgical approach is still debated in the literature and further investigations should be carried out, with meta-analytic strategies.

## Figures and Tables

**Table 1:** Descriptive statistics of study variables.

Variable, Measure	Unit	Summary
Demographic, anthropometric:		
Age, mean (SD)	Years	68.1 (10.4)
Male sex, n (%)	*n*	333 (44.8)
BMI, mean (SD)	Kg/m^2^	27.6 (4.4)
Outside Region, n (%)	*n*	107 (14.5)
Outside City, n (%)	*n*	378 (51.3)
Comorbidities:		
Diabetes Mellitus, n (%)	*n*	64 (8.7)
Arterial hypertension, n (%)	*n*	346 (46.8)
Dyslipidemia, n (%)	*n*	151 (20.4)
Heart failure, n (%)	*n*	6 (0.8)
Coronary disease, n (%)	*n*	14 (1.9)
COPD, n (%)	*n*	15 (2)
CKF, n (%)	*n*	88 (12.1)
Smokers, n (%)	*n*	98 (15.8)
Smoke in the past, n (%)	*n*	92 (14.8)
Preoperative laboratory values:		
BFG, mean (SD)	mg/dL	104.5 (19.2)
Hb, mean (SD)	g/dL	14(1.47)
WBC, mean (SD)	U/microL	6886.2 (1892.4)
ESR, mean (SD)	mm/h	32.4 (31.8)
CRP, mean (SD)	mg/dL	3.8 (3.1)
Creatinin, mean (SD)	mg/dL	0.86 (0.3)
eGFR (CKD-EPI), mean (SD)	mL/min	80.7 (16.8)
Perioperative factors:		
Operation time, mean (SD)	Minutes	78.3 (13.4)
Cemented stem, n (%)	*n*	31 (4.2)
Cemented cup, n (%)	*n*	34 (4.6)
Intraoperative blood loss, mean (SD)	ml	286.2 (156.8)
Hospitalization length (LOS):		
Hospitalization length, median (IQR)	days	4 (3,5)
Patients with LOS longer than the median, *n* (%)	*n*	251 (34.1)

SD = standard deviation, COPD = Chronic Obstructive Pulmonary Disease, CKF = chronic kidney failure, eGFR (CKD-EPI) = estimated glomerular filtration rate though CKD-EPI equation, BFG = blood fasting glucose, SC = Serum creatinine, WBC = white blood cell count, Hb = hemoglobin. ESR = Erythrocytes Sedimentation Rate, CRP = C-Reactive Protein, LOS = Length Of Stay.

**Table 2:** Charlson’s Comorbidity Index and ASA score.

Charlson’s Index	*n* (%)
0	623 (84.5)
1	26 (3.5)
2	80 (10.9)
3	8 (1.1)
4	0 (0)
ASA score	*n* (%)
1	26 (3.5)
2	703 (95.4)
3	8 (1.1)
4	0 (0)

**Table 3:** Surgical approach.

Approach	*n* (%)
Supine anterolateral	262 (35.7)
Supine inguinal	4 (0.54)
Supine direct lateral	310 (42.2)
Lateral decubitus posterolateral	159 (21.6)

**Table 4:** Regression models.

Variable	Univariate Negative Binomial		Multivariate Negative Binomial (Best Fit ^#^) Chi^2^ = 70.2		Multivariate Logistic Chi^2^ = 97.5	
	IRR [95% CI]	*p* Value	IRR [95% CI]	*p* Value	OR [95% CI]	*p* Value
Demographic, anthropometric:						
Age	1.00 [0.99, 1.01]	0.410				
Male sex	1.05 [0.96, 1.13]	0.236				
BMI	1.00 [0.99, 1.01]	0.311				
Outside Region	1.01 [0.91, 1.13]	0.716				
Outside City	0.98 [0.91, 1.06]	0.692				
Comorbidities:						
Diabetes Mellitus	0.97 [0.87, 1.1]	0.722				
Arterial hypertension	0.99 [0.91, 1.07]	0.78				
Dyslipidemia	1.01 [0.91, 1.12]	0.891				
Heart failure	0.73 [0.47, 1.15]	0.172				
Coronary disease	1.09 [0.84, 1.41]	0.492				
COPD	0.92 [0.77, 1.09]	0.367				
CKF	1.18 [1, 1.4]	0.041 *				
Smokers	0.99 [0.94, 1.05]	0.942				
Smoke in the past	0.97 [0.91, 1.03]	0.687				
CCI	1.07 [0.99, 1.15]	0.06				
CCI > 1	1.19 [1.06, 1.32]	0.002 *				
ASA	1.04 [0.87, 1.24]	0.654				
Preoperative laboratory values:						
BFG	1.00 [0.99, 1]	0.097				
Hb	0.98 [0.95, 1.01]	0.324				
WBC	1.00 [1, 1]	0.038 *				
VES	1.00 [0.99, 1.01]	0.571				
PCR	0.99 [0.94, 1.05]	0.927				
Creatinin	1.29 [1.01, 1.67]	0.041 *	1.33 [1.17, 1.53]	<0.001 *	1.43 [0.72, 2.84]	0.31
eGFR (CKD-EPI)	0.99 [0.99, 1]	0.165				
Perioperative factors:						
Operation time	1.003 [1, 1.004]	<0.001 *	1.002 [1, 1.003]	0.001 *	1.01 [1.003, 1.014]	<0.001 *
Cemented stem	1.17 [0.99, 1.38]	0.053				
Cemented cup	1.01 [0.88, 1.14]	0.91				
Intraoperative blood loss	1.001 [1, 1.001]	<0.001 *	1.0003 [1, 1.001]	0.04 *	1.001 [0.99, 1.002]	0.061
Anterolateral approach	1.25 [1.16, 1.36]	<0.001 *	1.22 [1.13, 1.33]	<0.001 *	3.9 [2.78, 5.7]	<0.001 *
Inguinal approach	0.86 [0.77, 0.98]	0.02 *				
Direct lateral approach	0.83 [0.77, 0.9]	<0.001 *				
Posterolateral approach	0.94 [0.86, 1.02]	0.155				

IRR = Incident Risk Ratio, OR = Odds Ratio, ^#^ after stepwise forward-back modeling, * Significant.

**Table 5:** Cut-off values.

Variable	Cut-Off	Youden’s Index
Creatinin	0.9 mg/dL	0.416
Intraoperative blood Loss	500 mL	
Operative time	89.2 min	

## Data Availability

The full dataset is available from the corresponding author, upon motivated request.
